# United States Travelers’ Concern about Zika Infection and Willingness to Receive a Hypothetical Zika Vaccine

**DOI:** 10.4269/ajtmh.17-0907

**Published:** 2018-04-23

**Authors:** Nadja A. Vielot, Lola Stamm, James Herrington, Linda Squiers, Bridget Kelly, Lauren McCormack, Sylvia Becker-Dreps

**Affiliations:** 1 Department of Family Medicine, School of Medicine, University of North Carolina at Chapel Hill, Chapel Hill, North Carolina;; 2 Department of Epidemiology, UNC Gillings School of Global Public Health, University of North Carolina at Chapel Hill, Chapel Hill, North Carolina;; 3 Department of Health Behavior, UNC Gillings School of Global Public Health, University of North Carolina at Chapel Hill, Chapel Hill, North Carolina;; 4 RTI International, Research Triangle Park, North Carolina

## Abstract

The ongoing Zika pandemic has affected many countries that are common travel destinations. We assessed the willingness to receive a prophylactic Zika virus (ZIKV) vaccine, currently under development, among travelers to areas with reported autochthonous ZIKV transmission. We surveyed United States (U.S.) residents aged 18–44 years who had ever heard of ZIKV and planned to travel to Florida and/or Texas (*N* = 420) or a U.S. territory or foreign country (*N* = 415) in 2017, using a nationally representative internet panel. Travelers to Florida and/or Texas reported less concern about ZIKV infection than travelers to other destinations (27% versus 36%, *P* = 0.01). Female sex, Hispanic ethnicity, discussing ZIKV with medical professionals, ZIKV risk perception, and self-efficacy for ZIKV prevention predicted concern about ZIKV infection in both groups. Travelers to Florida and/or Texas (43%) and other destinations (44%) were equally willing to receive a ZIKV vaccine. Hispanic ethnicity, discussing ZIKV with medical professionals, and concern about ZIKV infection predicted vaccine willingness in both groups. Likelihood of using existing ZIKV prevention methods, confidence in the U.S. government to prevent ZIKV spread, self-efficacy for ZIKV prevention, and knowledge about ZIKV symptoms further predicted vaccine willingness in travelers to other destinations. In multivariable analyses, only concern about ZIKV infection was associated with vaccine willingness in both groups (prevalence ratio [95% confidence interval]: Florida and/or Texas: 1.34 [1.06, 1.69]; other: 1.82 [1.44, 2.29]). Targeted communications can educate travelers, particularly travelers who are pregnant or may become pregnant, about ZIKV risk to generate ZIKV vaccine demand.

## INTRODUCTION

Since its detection in Brazil in early 2015, Zika virus (ZIKV) has spread rapidly throughout Latin America and the Caribbean. Nearly all countries in the region and two United States (U.S.) states and three territories have reported local (i.e., autochthonous) mosquito-borne transmission.^1^ Millions of Americans travel to these and other areas with autochthonous ZIKV transmission each year.^2^ Although ZIKV infection is asymptomatic in up to 80% of infected persons, several characteristics make ZIKV an important concern for U.S. travelers.^3^ ZIKV virus appears to be unique among flaviviruses in its ability to be transmitted from mother to fetus and to cause a constellation of severe birth defects now known as congenital Zika syndrome.^4–6^ Also, ZIKV infection has been found to be associated with the Guillain–Barré syndrome, a nervous system disorder that can cause symmetric muscle weakness and paralysis of the respiratory muscles.^7^ Furthermore, unlike other arboviruses, there is clear evidence that ZIKV can be transmitted sexually. Of 5,168 symptomatic cases of ZIKV reported in United States in 2016, 4,897 cases were travelers returning from ZIKV-affected areas, 224 cases were individuals presumably infected locally by mosquito bites (in Florida and Texas), and 45 cases were individuals infected by sexual transmission.^8^

In response to evolving knowledge about ZIKV, the U.S. Centers for Disease Control and Prevention (CDC) provided recommendations for travelers to areas with autochthonous ZIKV transmission.^9^ Travel notices for various countries in the Caribbean, Central and South America, Asia, and Africa remain in place 2 years after the first cases of microcephaly were reported in Brazil, and, until more can be done to prevent ZIKV infection and its acute and long-term sequelae, there is little hope that they will be lifted. Even as the initial epidemic wanes, ZIKV is likely to become endemic in many areas and remains a concern to local populations and travelers.^10^

Current strategies to prevent ZIKV infection focus largely on control of *Aedes aegypti* and *Aedes albopictus* mosquitoes, the known vectors; protection of the blood supply; and behavioral interventions, including the promotion of condom use during sex. No commercially available vaccine exists to prevent ZIKV infection. However, preclinical studies have demonstrated that multiple vaccine platforms (e.g., nucleic acid, inactivated virus, and recombinant vector-based) generate protective immunity in animal challenge models, and some of these vaccine candidates are currently in phase I human trials.^11–16^ RTI International and the University of North Carolina at Chapel Hill surveyed travelers living in U.S. states on their awareness and perceptions of ZIKV and its potential effects on their travel plans. The present study explored concern about ZIKV infection while traveling among U.S. travelers and their willingness to receive a hypothetical ZIKV vaccine.

## METHODS

We accessed a web-based, nationally representative panel of 50,000 adults living in the 50 U.S. states and Washington, DC from GfK Custom Research, LLC, described elsewhere.^17,18^ GfK emailed a link to a self-administered, web-based survey to 8,075 randomly selected panel members. This sample was confirmed to be representative of U.S. residents aged 18–44 years based on American Community Survey (ACS) data from 2015.^19^ Of these, 3,869 completed eligibility screening. Eligible respondents were aged 18–44 years; planned to travel between March and December 2017 to locations with a history of autochthonous ZIKV transmission, according to the CDC at the time of the survey; and reported having ever seen or heard any information about ZIKV.^20^ Qualifying travel destinations with ongoing autochthonous ZIKV transmission or a history of autochthonous transmission included U.S. states (Florida and Texas) and territories (American Samoa, the U.S. Virgin Islands, and Puerto Rico), the Caribbean Islands, Mexico, and various countries in Central and South America, Asia, and the Pacific Islands.^18^ Eligible respondents who consented to participate received periodic e-mail reminders to complete the survey in full. The survey was open between March 17 and April 4, 2017.

Respondents self-reported data on demographics, travel destinations, knowledge, attitudes, and practices around ZIKV and prevention of ZIKV, and willingness to receive a hypothetical ZIKV vaccine. We summarized the sample by demographics and travel patterns using descriptive statistics. We then conducted bivariate analyses to estimate predictors of 1) concern about being infected with ZIKV while traveling and 2) willingness to receive a ZIKV vaccine. Predictors of interest included demographic characteristics, pregnancy intentions, discussing ZIKV with a health-care professional, perceived likelihood of being bitten by a ZIKV-infected mosquito (i.e., risk perception for ZIKV infection), confidence in the U.S. federal government to prevent ZIKV spread, confidence in oneself to understand ZIKV transmission and prevention (i.e., self-efficacy for ZIKV prevention), willingness to use existing ZIKV prevention methods, and knowledge of ZIKV. Concern about ZIKV infection was additionally assessed as a predictor of ZIKV vaccine willingness. Confidence in the U.S. federal government to control ZIKV, self-efficacy for ZIKV prevention, and concern about ZIKV infection were measured using three-point Likert scales. Willingness to use existing prevention methods was measured using four-point Likert scales for nine different prevention methods (e.g., using a mosquito repellant and wearing long sleeves and long pants) and by indicating some likelihood of using at least one of these methods. Next, we summarized respondents’ knowledge about ZIKV in three categories: transmission routes, symptoms, and ZIKV transmission and prevention in pregnancy (Supplemental Appendix). Knowledge scores represent the percentage of correct responses in each category, and the score distribution was divided into three groups with equal probabilities, ranked as high, medium, or low.

We used the Mantel–Haenszel χ^2^ test to identify bivariate predictors of concern about ZIKV infection and willingness to receive a ZIKV vaccine. Fisher’s exact test was used in the event of small cell sizes (*N* < 5). Finally, we used log-binomial multivariable regression to estimate prevalence ratios (PR) and 95% confidence intervals for the association between selected independent variables and an individual’s willingness to receive a ZIKV vaccine, the dependent variable. Independent variables that were significantly associated at the α = 0.05 level in bivariate analyses with willingness to receive a ZIKV vaccine were included in the multivariable model. The association of pregnancy intentions with vaccine willingness was estimated in a women-only analysis, identifying model covariates using the same methods described previously. Correlated covariates were dropped from the final models to achieve parsimony and model convergence.

Given that Florida and Texas did not have ongoing autochthonous ZIKV transmission at the time of the survey, we stratified all analyses by destination (Florida and/or Texas versus other destinations) to account for differences in willingness to receive a ZIKV vaccine by incidence of ZIKV transmission in intended travel destinations. We also weighted individuals in the final sample to be more representative of the U.S. population aged 18–44 years in 2015, as described elsewhere.^18^ All analyses were conducted using SAS version 9.4 (SAS Institute, Cary, NC).

## RESULTS

**Figure 1.**
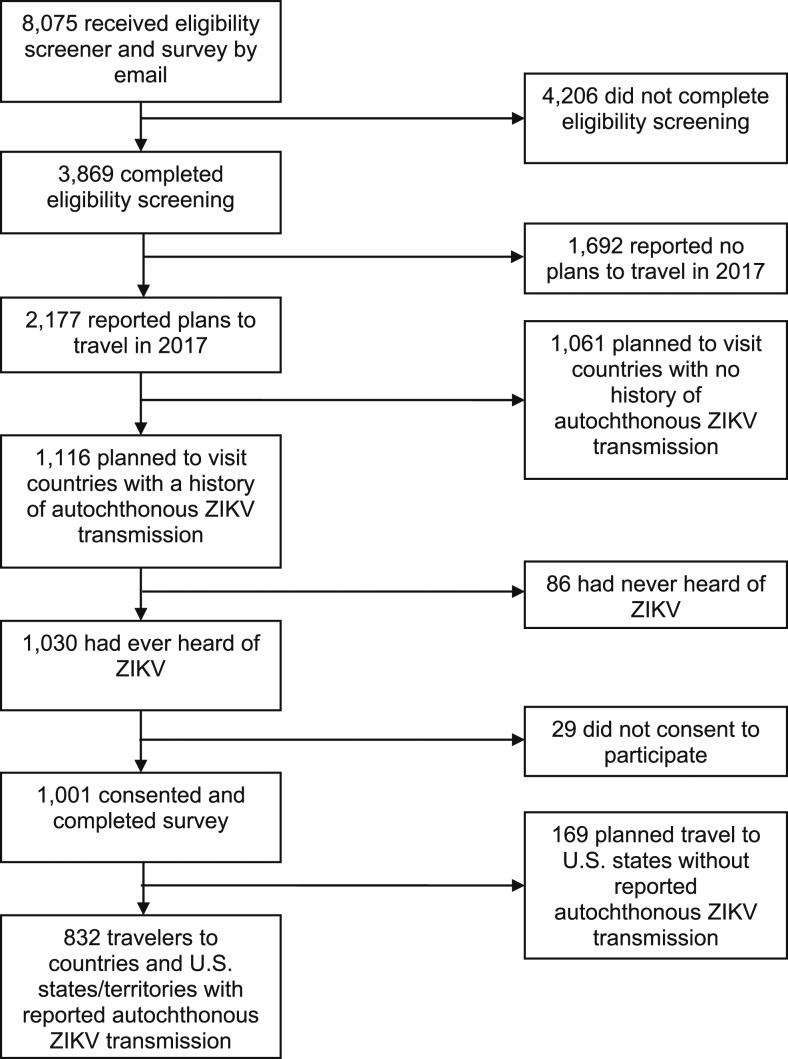
Selection of final analytic sample from United States (U.S.) residents contacted from the GfK KnowledgePanel database.^17^

Of 8,075 panel members screened, 13.8% (*N* = 1,116) planned to travel in 2017 to countries and regions with reported autochthonous ZIKV transmission. Most respondents (93%, *N* = 1,030) had ever heard of ZIKV, and 1,001 consented to participate in the survey. We eliminated 169 respondents who planned to travel exclusively to U.S. states with no history of reported autochthonous ZIKV transmission, yielding a final analytic sample of 832 travelers (Figure 1). The sample size after weighting was *N* = 835. The weighted sample was representative of the general U.S. population based on 2015 ACS data with respect to age, race/ethnicity, and urban residence.

One-half of respondents (*N* = 420) reported travel exclusively to Florida and/or Texas, whereas one-half (*N* = 415) reported travel exclusively to U.S. territories or foreign countries (Table 1). Respondents overall were predominantly female (56%), aged 25 years or older (88%), and White (73%). The median annual household income range was $60,000–$84,000 (Table 1). Thirty-eight percent of respondents had a bachelor’s degree or higher and a minority of travelers were full-time (13%) or part-time (10%) college students. Most respondents (82%) planned to travel to only one destination, which was primarily Florida and/or Texas. Overall, respondents primarily reported travel for leisure, vacation, or adventure (55%), followed by visiting friends or relatives (36%) (Table 1). Respondents planning travel to destinations outside of U.S. states tended to be older, more likely to identify as Hispanic, and more likely to plan travel for leisure, vacation, or adventure (Table 1).

**Table 1:** Characteristics of survey respondents traveling to ZIKV-affected areas in 2017, by travel destination

	Total (weighted, *N* = 835)	Florida and/or Texas (weighted, *N* = 420)	Other destinations (weighted, *N* = 415)
	*n* (%)	*n* (%)	*n* (%)
Sex			
Female	471 (56.4)	240 (57.1)	231 (55.6)
Male	364 (43.6)	180 (42.9)	184 (44.4)
Age group (years)			
18–24	187 (22.4)	98 (23.3)	89 (21.4)
25–34	328 (39.3)	179 (42.6)	150 (36.0)
35–44	320 (38.3)	143 (34.1)	177 (42.6)
Race			
White	607 (72.7)	320 (76.2)	288 (69.3)
Black/African American	125 (15.0)	65 (15.4)	61 (14.6)
American Indian or Alaskan Native, Native Hawaiian or Pacific Islander	14 (1.6)	2 (0.4)	12 (2.8)
Asian	60 (7.2)	24 (5.7)	36 (8.7)
Other	29 (3.5)	10 (2.4)	19 (4.6)
Ethnicity			
Not Spanish, Hispanic, or Latino	616 (73.8)	345 (82.1)	271 (65.3)
Mexican, Mexican-American, and Chicano	114 (13.7)	30 (7.2)	84 (20.3)
Puerto Rican	31 (3.7)	14 (3.4)	17 (4.1)
Cuban, Cuban American	19 (2.3)	9 (2.2)	10 (2.4)
Other Spanish, Hispanic, or Latino group	55 (6.6)	21 (5.1)	22 (8.0)
Residence in ZIKV-affected states			
Florida or Texas	190 (22.8)	93 (22.1)	97 (23.5)
Other state	645 (77.2)	327 (77.9)	318 (76.5)
Household income level (Quintiles)			
$0–29,999	100 (12.0)	51 (12.1)	48 (11.7)
$30,000–59,999	191 (22.9)	95 (22.6)	96 (23.2)
$60,000–84,999	158 (18.9)	87 (20.6)	71 (17.1)
$85,000–124,999	186 (22.2)	96 (22.9)	90 (21.6)
$125,000+	201 (24.1)	91 (21.8)	110 (26.5)
Education level			
Less than high school	80 (9.6)	39 (9.4)	40 (9.7)
High school	155 (18.6)	74 (17.6)	81 (19.4)
Some college	283 (33.9)	144 (34.4)	139 (33.4)
Bachelor’s degree or higher	318 (38.1)	162 (38.6)	156 (37.5)
Current college student			
No	650 (77.8)	330 (78.6)	320 (77.1)
Yes, full-time	105 (12.6)	49 (11.7)	55 (13.3)
Yes, part-time	80 (9.6)	41 (9.7)	40 (9.6)
Number of travel destinations*			
One destination	682 (81.7)	420 (100.0)	262 (63.1)
Two or more destinations	153 (18.3)	–	153 (36.9)
Purpose of trip			
Business	43 (5.2)	27 (6.5)	16 (3.9)
Leisure, vacation, or adventure	461 (55.3)	221 (52.7)	240 (58.0)
Visiting friends or relatives	297 (35.6)	162 (38.5)	135 (32.7)
Other†	32 (3.9)	10 (2.3)	23 (5.4)

	Missing (*N* = 2)		Missing (*N* = 2)
Travel destinations†‡			
U.S. state (Florida and/or Texas)	527 (63.1)	420 (100.0)	107 (25.8)
U.S. territory (U.S., Virgin Islands, American Samoa, and Puerto Rico)	64 (7.7)	–	64 (15.4)
Caribbean Islands	194 (23.2)	–	194 (46.7)
Mexico	189 (22.7)	–	189 (45.5)
Central/South America	52 (6.2)	–	52 (12.5)
Asia/Pacific Islands	20 (2.4)	–	20 (4.8)

U.S. = United States; ZIKV = Zika virus.

* Travel to a U.S. state (i.e., Florida and Texas) and travel to a U.S. territory (i.e., Puerto Rico, American Samoa, or U.S. Virgin Islands) are each considered a single destination.

† Other includes providing or receiving medical care, research or education, and mission or nonmedical service.

‡ Total percent may be greater than 100% because of multiple destinations reported.

Among all respondents, 63% reported travel to Florida and/or Texas, 8% reported travel to a U.S. territory, 3% reported travel to the Caribbean Islands, 23% reported travel to Mexico, 6% reported travel to Central and/or South America, and 2% reported travel to Asia and/or the Pacific Islands (Table 1). Among 527 respondents traveling to U.S. states, 60.2% reported travel to Florida and 24.6% reported travel to Texas.

Among 833 respondents, 262 (31%) were somewhat or very concerned about becoming infected with ZIKV while traveling: 27% of travelers to Florida and/or Texas and 36% of travelers to other destinations (*P* = 0.01). The following predictors of concern about ZIKV infection were common to both groups of respondents: Hispanic ethnicity (Florida and/or Texas: *P* < 0.0001; other destinations: *P* = 0.03), discussing ZIKV with a medical professional (Florida and/or Texas: *P* = 0.008; other destinations: *P* = 0.001), risk perception for ZIKV infection (Florida and/or Texas: *P* = 0.0006; other destinations: *P* < 0.0001), and self-efficacy for ZIKV prevention (Florida and/or Texas: *P* = 0.05; other destinations: *P* = 0.002) (Table 2). Female sex (*P* = 0.02), confidence in the U.S. government to prevent ZIKV spread (*P* = 0.05), and low knowledge of ZIKV transmission patterns (*P* = 0.04) and symptoms (*P* = 0.001) were significantly associated with concern about ZIKV infection among travelers to Florida and/or Texas only (Table 2). Among respondents traveling to destinations outside of U.S. states, those who were likely to use an existing ZIKV prevention method were three times as likely to be concerned about ZIKV (*P* = 0.03) (Table 2).

**Table 2:** Bivariate predictors of concern about ZIKV infection among travelers to ZIKV-affected areas in 2017, by travel destination

Florida and/or Texas (weighted, *N* = 420)			Other destinations (weighted, *N* = 415)		
	Concerned *n* (%)	P*		Concerned *n* (%)	P*
Sex			Sex		
Female (*N* = 240)	77 (31.9)	**0.02**	Female (*N* = 231)	91 (39.3)	0.07
Male (*N* = 180)	39 (21.5)	–	Male (*N* = 183)	56 (30.6)	–
			Missing (*N* = 2)		
Age group			Age group		
18–33 years (*N* = 259)	65 (25.1)	0.2	18–33 years (*N* = 223)	86 (38.4)	0.2
34–44 years (*N* = 161)	50 (31.2)	–	34–44 years (*N* = 190)	61 (32.1)	–
			Missing (*N* = 2)		
Ethnicity			Ethnicity		
Not Hispanic (*N* = 345)	78 (23.1)	**< 0.0001**	Not Hispanic (*N* = 270)	86 (31.7)	**0.03**
Hispanic (*N* = 75)	36 (47.5)	–	Hispanic (*N* = 143)	61 (42.6)	–
			Missing (*N* = 2)		
Residence in ZIKV-affected states			Residence in ZIKV-affected states		
Florida or Texas (*N* = 92)	31 (33.7)	0.1	Florida or Texas (*N* = 97)	34 (35.3)	1.0
Other (*N* = 327)	84 (25.7)	–	Other (*N* = 316)	112 (35.5)	–
			Missing (*N* = 2)		
Currently pregnant (*N* = 240 women)			Currently pregnant (*N* = 231 women)		
Yes (*N* = 11)	4 (31.4)	1.0	Yes (*N* = 0)	0 (0.0)	–
No (*N* = 227)	73 (32.2)	–	No (*N* = 229)	90 (39.5)	–
Missing (*N* = 2)			Missing (*N* = 2)		
Planning to become pregnant (*N* = 240 women)			Planning to become pregnant (*N* = 231 women)		
Yes (*N* = 13)	7 (53.2)	0.09	Yes (*N* = 12)	6 (51.1)	0.4
No (*N* = 213)	66 (30.9)	–	No (*N* = 214)	84 (39.2)	–
Missing (*N* = 13)			Missing (*N* = 5)		
Discussed ZIKV with a medical professional			Discussed ZIKV with a medical professional		
Yes (*N* = 35)	16 (47.2)	**0.008**	Yes (*N* = 41)	24 (58.3)	**0.001**
No (*N* = 378)	98 (26.0)	–	No (*N* = 372)	123 (32.9)	–
Missing (*N* = 7)			Missing (*N* = 2)		
Likelihood of being bitten by a ZIKV-infected mosquito			Likelihood of being bitten by a ZIKV-infected mosquito		
Not at all likely (*N* = 215)	43 (20.0)	**0.0006**	Not at all likely (*N* = 185)	44 (23.6)	**< 0.0001**
Somewhat to very likely (*N* = 194)	68 (35.1)	–	Somewhat to very likely (*N* = 208)	90 (43.2)	–
Missing (*N* = 11)			Missing (*N* = 2)		
Likelihood of using existing ZIKV prevention methods†			Likelihood of using existing ZIKV prevention methods†		
Not at all likely (*N* = 13)	1 (10.1)	0.1	Not at all likely (*N* = 20)	2 (12.2)	**0.03**
Somewhat to very likely (*N* = 400)	113 (28.1)	–	Somewhat to very likely (*N* = 389)	142 (36.5)	–
Missing (*N* = 7)			Missing (*N* = 6)		
Confidence in the U.S. government’s ability to prevent ZIKV spread			Confidence in the U.S. government’s ability to prevent ZIKV spread		
Unconfident (*N* = 151)	34 (22.8)	**0.05**	Unconfident (*N* = 139)	51 (34.9)	0.8
Neither confident nor unconfident (*N* = 142)	50 (34.9)	–	Neither confident nor unconfident (*N* = 157)	52 (35.8)	–
Confident (*N* = 115)	28 (24.6)	–	Confident (*N* = 115)	43 (29.3)	–
Missing (*N* = 12)			Missing (*N* = 4)		
Confidence in your ability to protect yourself and your family from ZIKV			Confidence in your ability to protect yourself and your family from ZIKV		
Unconfident (*N* = 60)	16 (26.2)	**0.05**	Unconfident (*N* = 61)	31 (51.1)	**0.002**
Neither confident nor unconfident (*N* = 132)	46 (35.2)	–	Neither confident nor unconfident (*N* = 135)	35 (25.7)	–
Confident (*N* = 216)	50 (23.1)	–	Confident (*N* = 215)	81 (37.4)	–
Missing (*N* = 12)			Missing (*N* = 4)		
ZIKV transmission knowledge score‡			ZIKV transmission knowledge score‡		
Low (*N* = 104)	39 (37.8)	**0.04**	Low (*N* = 99)	38 (38.7)	0.1
Medium (*N* = 191)	46 (24.2)	–	Medium (*N* = 173)	52 (30.2)	–
High (*N* = 93)	24 (25.5)	–	High (*N* = 109)	45 (41.0)	–
Missing (*N* = 32)			Missing (*N* = 34)		
ZIKV symptoms knowledge score‡			ZIKV symptoms knowledge score‡		
Low (*N* = 127)	54 (42.7)	**0.001**	Low (*N* = 105)	48 (45.6)	0.4
Medium (*N* = 81)	23 (27.9)	–	Medium (*N* = 83)	30 (35.8)	–
High (*N* = 66)	12 (18.0)	–	High (*N* = 74)	32 (43.1)	–
Missing (*N* = 146)			Missing (*N* = 152)		
ZIKV in pregnancy knowledge score‡			ZIKV in pregnancy knowledge score‡		
Low (*N* = 135)	43 (32.0)	0.6	Low (*N* = 135)	43 (32.0)	0.4
Medium (*N* = 189)	72 (38.3)	–	Medium (*N* = 189)	72 (38.3)	–
High (*N* = 24)	7 (30.5)	–	High (*N* = 24)	7 (30.5)	–
Missing (*N* = 74)			Missing (*N* = 67)		

ZIKV = Zika virus. Bold values are statistically significant at α = 0.05 level.

* *P* value represents χ^2^ tests for differences in vaccine willingness vs. unwillingness by levels of dependent variables, within each stratum of travel destination. Fisher’s exact test was used for comparisons in which cell sizes were < 5.

† Prevention methods including using a mosquito repellent; wearing long sleeves and long pants; staying in places with air conditioning, window screens, or door screens; treating clothing with permethrin; avoiding casual contact with others; sleeping under a bednet; staying sober to avoid casual sex with persons traveling to ZIKV-affected areas; abstaining from sex with persons traveling to ZIKV-affected areas; and using condoms when having sex with persons traveling to ZIKV-affected areas.

‡ Low, medium, and high knowledge scores are determined based on tertiles for the percentage of correct responses in each category. Transmission: low = 0–66%, medium = 67–83%, high = 84–100%. Symptoms: low = 0–50%, medium = 63–74%, high = 75–100%. Pregnancy: low = 0–74%, medium = 75–99%, high = 100%.

Respondents traveling to Florida and/or Texas (43%) and other destinations (44%) were equally willing to receive a ZIKV vaccine. Hispanic ethnicity (Florida and/or Texas: *P* < 0.0001; other destinations: *P* = 0.03), discussing ZIKV with a medical professional (Florida and/or Texas: *P* < 0.0001; other destinations: *P* = 0.005), and concern about ZIKV infection while traveling (both *P* < 0.0001) were significant positive predictors of willingness to receive a ZIKV vaccine in both groups (Table 3). Female sex was associated with greater willingness to receive a ZIKV vaccine among travelers to Florida and/or Texas (*P* = 0.02), whereas likelihood of using existing ZIKV prevention methods (*P* = 0.001), self-efficacy for ZIKV prevention (*P* = 0.0006), and low knowledge of ZIKV symptoms (*P* = 0.02) were associated with greater willingness to receive a ZIKV vaccine among travelers to destinations outside of U.S. states (Table 3).

**Table 3:** Bivariate predictors of willingness to receive a ZIKV vaccine among travelers to ZIKV-affected areas in 2017, by travel destination

Florida and/or Texas (weighted, *N* = 420)			Other destinations (weighted, *N* = 415)		
	Willing *n* (%)	P*		Willing *n* (%)	P*
Sex			Sex		
Female (*N* = 235)	113 (48.3)	**0.02**	Female (*N* = 230)	110 (47.8)	0.09
Male (*N* = 179)	66 (37.0)		Male (*N* = 181)	71 (39.4)	
Missing (*N* = 6)			Missing (*N* = 4)		
Age group			Age group		
18–33 years (*N* = 255)	118 (46.2)	0.2	18–33 years (*N* = 223)	106 (47.7)	0.1
34–44 years (*N* = 158)	62 (39.0)		34–44 years (*N* = 189)	75 (39.8)	
Missing (*N* = 6)			Missing (*N* = 4)		
Ethnicity			Ethnicity		
Not Hispanic (*N* = 340)	131 (38.4)	**< 0.0001**	Not Hispanic (*N* = 270)	109 (40.3)	**0.03**
Hispanic (*N* = 74)	49 (66.4)		Hispanic (*N* = 141)	73 (51.3)	
Missing (*N* = 6)			Missing (*N* = 4)		
Residence in ZIKV-affected states			Residence in ZIKV-affected states		
Florida or Texas (*N* = 92)	41 (45.2)	0.7	Florida or Texas (*N* = 97)	41 (41.9)	0.6
Other (*N* = 322)	138 (42.9)	–	Other (*N* = 315)	141 (44.8)	
Missing (*N* = 6)			Missing (*N* = 4)		
Currently pregnant (*N* = 240 women)			Currently pregnant (*N* = 231 women)		
Yes (*N* = 11)	7 (69.0)	0.2	Yes (*N* = 0)	0 (0.0)	–
No (*N* = 222)	104 (46.9)		No (*N* = 229)	110 (48.1)	
Missing (*N* = 7)			Missing (*N* = 2)		
Planning to become pregnant (*N* = 240 women)			Planning to become pregnant (*N* = 231 women)		
Yes (*N* = 13)	8 (63.6)	0.2	Yes (*N* = 12)	0 (0.0)	0.08
No (*N* = 209)	96 (45.8)		No (*N* = 214)	110 (48.06)	
Missing (*N* = 18)			Missing (*N* = 4)		
Discussed ZIKV with a medical professional			Discussed ZIKV with a medical professional		
Yes (*N* = 34)	26 (77.1)	**< 0.0001**	Yes (*N* = 41)	27 (64.9)	**0.005**
No (*N* = 375)	151 (40.3)		No (*N* = 371)	155 (41.8)	
Missing (*N* = 11)			Missing (*N* = 4)		
Likelihood of being bitten by a ZIKV-infected mosquito			Likelihood of being bitten by a ZIKV-infected mosquito		
Not at all likely (*N* = 214)	90 (42.2)	0.8	Not at all likely (*N* = 184)	71.4 (38.8)	0.08
Somewhat to very likely (*N* = 191)	83 (43.7)		Somewhat to very likely (*N* = 207)	98.8 (47.7)	
Missing (*N* = 15)			Missing (*N* = 24)		
Likelihood of using existing ZIKV prevention methods†			Likelihood of using existing ZIKV prevention methods†		
Not at all likely (*N* = 13)	5 (37.1)	0.6	Not at all likely (*N* = 20)	2 (8.6)	**0.001**
Somewhat to very likely (*N* = 397)	173 (43.7)		Somewhat to very likely (*N* = 388)	177 (45.6)	
Missing (*N* = 10)			Missing (*N* = 8)		
Confidence in the U.S. government’s ability to prevent ZIKV spread			Confidence in the U.S. government’s ability to prevent ZIKV spread		
Unconfident (*N* = 151)	62 (41.4)	0.09	Unconfident (*N* = 138)	61 (43.8)	**0.02**
Neither confident nor unconfident (*N* = 141)	57 (40.1)		Neither confident nor unconfident (*N* = 156)	58 (37.0)	
Confident (*N* = 112)	59 (52.8)		Confident (*N* = 115)	62 (53.9)	
Missing (*N* = 16)			Missing (*N* = 6)		
Confidence in your ability to protect yourself and your family from ZIKV			Confidence in your ability to protect yourself and your family from ZIKV		
Unconfident (*N* = 60)	29 (47.9)	0.2	Unconfident (*N* = 61)	27 (43.7)	**0.0006**
Neither confident nor unconfident (*N* = 132)	49 (37.3)		Neither confident nor unconfident (*N* = 42)	42 (31.1)	
Confident (*N* = 213)	101 (47.3)		Confident (*N* = 112)	112 (52.3)	
Missing (*N* = 16)			Missing (*N* = 6)		
Concern about ZIKV infection while traveling			Concern about ZIKV infection while traveling		
Not at all concerned (*N* = 299)	112 (37.4)	–	Not at all concerned (*N* = 267)	85 (31.8)	**< 0.0001**
Somewhat or very concerned (*N* = 114)	67 (59.3)	**< 0.0001**	Somewhat or very concerned (*N* = 145)	97 (66.9)	
Missing (*N* = 7)			Missing (*N* = 4)		
ZIKV transmission knowledge score‡			ZIKV transmission knowledge score‡		
Low (*N* = 104)	47 (45.0)	0.9	Low (*N* = 98)	45 (45.7)	0.4
Medium (*N* = 189)	83 (44.0)		Medium (*N* = 172)	71 (41.5)	
High (*N* = 91)	42 (46.3)		High (*N* = 109)	54 (49.8)	
Missing (*N* = 36)			Missing (*N* = 36)		
ZIKV symptoms knowledge score‡			ZIKV symptoms knowledge score‡		
Low (*N* = 125)	64 (51.7)	0.7	Low (*N* = 105)	66 (63.3)	**0.02**
Medium (*N* = 80)	37 (45.9)		Medium (*N* = 36)	35.9 (44.0)	
High (*N* = 64)	31 (48.3)		High (*N* = 74)	34.9 (46.9)	
Missing (*N* = 151)			Missing (*N* = 154)		
ZIKV in pregnancy knowledge score‡			ZIKV in pregnancy knowledge score‡		
Low (*N* = 117)	48 (41.0)	0.4	Low (*N* = 135)	58 (43.0)	0.6
Medium (*N* = 186)	91 (48.8)		Medium (*N* = 187)	90 (48.4)	
High (*N* = 39)	18 (45.7)		High (*N* = 24)	12 (50.8)	
Missing (*N* = 78)			Missing (*N* = 69)		

ZIKV = Zika virus. Bold values are statistically significant at α = 0.05 level.

* *P* value represents χ^2^ tests for differences in vaccine willingness by levels of dependent variables. Fisher’s exact test was used for comparisons in which cell sizes were < 5.

† Prevention methods including using a mosquito repellent; wearing long sleeves and long pants; staying in places with air conditioning, window screens, or door screens; treating clothing with permethrin; avoiding casual contact with others; sleeping under a bednet; staying sober to avoid casual sex with persons traveling to ZIKV-affected areas; abstaining from sex with persons traveling to ZIKV-affected areas; and using condoms when having sex with persons traveling to ZIKV-affected areas.

‡ Low, medium, and high knowledge scores are determined based on tertiles for the percentage of correct responses in each category. Basic knowledge: low = 0–50%, medium = 51–75%, high = 76–100%. Transmission: low = 0–66%, medium = 67–83%, high = 84–100%. Symptoms: low = 0–50%, medium = 63–74%, high = 75–100%. Pregnancy: low = 0–74%, medium = 75–99%, high = 100%.

Table 4 displays the results of multivariable models for predictors of willingness to receive a ZIKV vaccine, including covariates from Table 3 that were significant at the 0.05 alpha level in each stratum of travel destination. Among respondents traveling to Florida and/or Texas, Hispanic ethnicity (PR: 1.38 [1.07, 1.78]), discussing ZIKV with a medical professional (PR: 1.42 [1.12, 1.81]), and concern about ZIKV infection while traveling (PR: 1.34 [1.06, 1.69]) remained significantly associated with willingness to receive a ZIKV vaccine (Table 4). Among travelers to destinations outside of U.S. states, concern about ZIKV infection (PR: 1.82 [1.44, 2.29]) was the only positive predictor of willingness to receive a ZIKV vaccine. Knowledge of ZIKV symptoms was a negative predictor of willingness to receive a ZIKV vaccine (medium versus low knowledge: PR: 0.76 [0.58, 0.98]; high versus low knowledge: PR: 0.77 [0.61, 0.98]) (Table 4).

**Table 4:** Multivariable predictors of willingness to receive a ZIKV vaccine among travelers to ZIKV-affected areas in 2017, by travel destination

	Florida and/or Texas (weighted, *N* = 420)	Other destinations (weighted, *N* = 415)
	Prevalence ratio (95% Confidence interval)*	Prevalence ratio (95% Confidence interval)*
Sex		
Female	1.0 (ref)	–
Male	0.85 (0.67, 1.07)	–
Ethnicity		
Non-Hispanic	1.0 (ref)	1.0 (ref)
Hispanic	**1.38 (1.07, 1.78)**	0.94 (0.78, 1.13)
Discussed ZIKV with a medical professional		
No	1.0 (ref)	1.0 (ref)
Yes	**1.42 (1.12, 1.81)**	1.09 (0.89, 1.35)
Concern about ZIKV infection while traveling		
Not at all concerned	1.0 (ref)	1.0 (ref)
Somewhat or very concerned	**1.34 (1.06, 1.69)**	**1.82 (1.44, 2.29)**
Confidence in your ability to protect yourself and your family from ZIKV		
Unconfident	–	1.0 (ref)
Neither confident nor unconfident	–	0.84 (0.58, 1.21)
Confident	–	1.11 (0.85, 1.44)
ZIKV symptoms knowledge score		
Low	–	1.0 (ref)
Medium	–	**0.76 (0.58, 0.98)**
High	–	**0.77 (0.61, 0.98)**

ZIKV = Zika virus. Bold values are statistically significant at α = 0.05 level.

* Models include modifiable variables that were statistically significantly associated with willingness to receive a ZIKV vaccine at the 0.05 level. All prevalence ratios are adjusted for all other variables in the model.

Among 231 women who were not pregnant at the time of the survey and planned to travel outside of U.S. states, planning a pregnancy was significantly associated with increased willingness to receive a ZIKV vaccine (PR: 1.60 [1.12, 2.27]). However, after adjustment for Hispanic ethnicity, discussing ZIKV with a medical professional, self-efficacy for ZIKV prevention, and concern about ZIKV infection while traveling, this association was no longer statistically significant (PR: 1.35 [0.93, 1.93]). Concern about ZIKV was the only remaining significant predictor of willingness to receive a ZIKV vaccine among women traveling outside of U.S. states (PR: 1.36 [1.03, 1.80]).

## DISCUSSION

This is the first published report of willingness to receive a hypothetical ZIKV vaccine among residents of U.S. states traveling to areas with a history of reported autochthonous ZIKV transmission. Nearly one-half of respondents were willing to receive a ZIKV vaccine. Demographic factors, risk perception for ZIKV infection, and self-efficacy for preventing ZIKV infection appeared to influence concern about exposure to ZIKV infection while traveling. After controlling for significant bivariate predictors of ZIKV vaccine willingness, concern about ZIKV infection while traveling was the only positive predictor of ZIKV vaccine willingness shared by travelers to Florida and/or Texas and to destinations outside of U.S. states. Although the peak of the ZIKV epidemic in the Western Hemisphere appears to have subsided, ZIKV endemicity remains a concern as the scientific community explores potential long-term sequelae of ZIKV infections in infected individuals and infants exposed in utero, including neurological, developmental, and other manifestations.^10,21–23^ A vaccine could effectively prevent ZIKV, provided there is sufficient interest to promote enrollment in vaccine trials and willingness to receive a licensed vaccine. Concern about ZIKV infection might be a key factor in motivating individuals to receive a ZIKV vaccine, regardless of their intended travel destinations.^24^

Prior research has focused on concern about infection as a predictor of infection control behaviors. Similar to our findings, a study of Malaysian adults found that increased worry (concern) about ZIKV relative to dengue was positively associated with practicing mosquito control after declaring ZIKV a public health emergency.^25^ A Canadian study of H1N1 influenza vaccination found that concern about H1N1, but not perceived risk of H1N1, increased the odds of planning to vaccinate.^26^ Risk perception in our study, indicated by perceived likelihood of being bitten by a ZIKV-infected mosquito, was not associated with increased willingness to receive a ZIKV vaccine. However, respondents with some ZIKV risk perception were nearly twice as likely to express concern about ZIKV infection, suggesting that risk perception has an indirect effect on willingness to receive a ZIKV vaccine through concern. This finding is supported by prior studies suggesting that perceived concern and perceived risk mediate one another to influence influenza vaccination.^27,28^

Among travelers to destinations outside of U.S. states, concern about ZIKV infection confounded the relationships between ethnicity, self-efficacy, and preventive behaviors and willingness to receive a ZIKV vaccine. By contrast, we found multiple independent positive predictors of willingness to receive a ZIKV vaccine among travelers to Florida and/or Texas. Hispanic travelers to Florida and/or Texas reported greater willingness to receive a ZIKV vaccine than those traveling outside of U.S. states. Post hoc analyses found that Hispanic respondents traveling outside of U.S. states were significantly more likely than non-Hispanics to be visiting family or friends (52% versus 22%, *P* < 0.0001), whereas this association was not found among travelers to Florida and/or Texas. We hypothesize that Hispanic respondents traveling to visit friends or relatives (possibly in their countries of origin) may be less concerned about acquiring travel-related illnesses. This finding would be consistent with prior findings of decreased adoption of preventive behaviors among travelers visiting friends and relatives in their countries of origin.^29,30^ Also, among travelers to Florida and/or Texas, having discussed ZIKV with a medical professional was associated with an individual’s willingness to receive a ZIKV vaccine. Given the cross-sectional nature of our study, it is unclear whether concern about ZIKV infection led travelers to discuss it with their medical providers or the reverse. However, only 10% of respondents reported discussing ZIKV with a medical professional ahead of their travel.^18^ Because only a minority of travelers discuss their travel plans with a medical provider, it may also be important to provide the vaccine outside of medical offices, such as in pharmacies and travel clinics, to increase access, similar to the availability of routine seasonal influenza immunization in the U.S.

Although the number of women who were pregnant or planning pregnancy was small, pregnancy intentions did not influence ZIKV vaccine willingness. By contrast, a study on willingness to receive a ZIKV vaccine among 989 pregnant women in Malaysia found 94% willingness to be vaccinated, and greater willingness to receive a ZIKV vaccine if it were recommended by a physician, friend, or relative.^31^ In a study of 408 pregnant women in Atlanta, GA, 73% of women sought ZIKV information from the CDC’s website compared with < 20% who sought information from their provider’s website, and a majority preferred an informational brochure or e-mail over information on the provider’s website.^32^ Providers should take advantage of these communication tools to educate their pregnant patients about ZIKV and the need for prevention. Furthermore, should a ZIKV vaccine become available, providers will need to actively inquire about their patients’ travel plans and offer the vaccine to patients visiting at-risk areas, particularly if they are pregnant or planning pregnancy.

Respondents reported similar levels of willingness to receive a ZIKV vaccine across all levels of knowledge with respect to ZIKV transmission and prevention in pregnancy, suggesting that knowledge of ZIKV is not sufficient to generate interest in ZIKV vaccination. Given that ZIKV symptoms outside of pregnancy are generally mild and self-limited, increased awareness of ZIKV symptoms, transmission, and prevention methods may actually reduce vaccine interest, as suggested by our findings among travelers outside of U.S. states. However, the cross-sectional nature of this study makes it difficult to know whether interest in preventing ZIKV fuels information-seeking and increased knowledge, or the reverse. These findings indicate that knowledge can influence vaccine willingness in various ways, and future studies should implement qualitative methods to identify specific motivations for vaccination.

Study limitations include exclusion of individuals who had never before heard of ZIKV. It is unknown if travelers who had never heard of ZIKV would have had different perceptions of ZIKV vaccination compared with those who had, or if awareness of ZIKV resulted from planning travel to at-risk locations. In addition, our sample included travelers to Florida and/or Texas who had a history of autochthonous transmission but no ongoing transmission at the time of the study. However, our stratified analysis allowed us to assess differences in predictors of ZIKV vaccine willingness between travelers to low- and high-transmission areas. We retained in the sample residents of Florida and/or Texas who reported travel within those states to improve statistical power, although these travelers may have different travel motivations and behaviors from those traveling outside of their home states. Furthermore, our study sample had a small number of individuals who were pregnant or planning pregnancy. However, we included a relatively large number of individuals of reproductive age, which yields useful results given that nearly one-half of all pregnancies in the U.S. are unplanned.^33^ Finally, whereas the GfK KnowledgePanel from which the analytic sample was drawn is representative of the general U.S. population, our sample of travelers had a higher representation of wealthy, college-educated, Southern, and bilingual Hispanic respondents. These demographics may represent the traveling population, rather than the general U.S. population. Strengths of this study include a nationwide sample and a stratified analysis to distinguish predictors of ZIKV vaccine willingness between travelers to areas with low and high risk of ZIKV transmission.

Additional research is needed to explore predictors of willingness to receive a ZIKV vaccine that were not assessed here. It is also important to understand drivers of concern about ZIKV infection to help travelers effectively address their concern, by either improving knowledge about ZIKV or offering recommendations for effective ZIKV prevention. A recent study of psychological predictors of anxiety around ZIKV infection found that contamination cognition severity, or the tendency to overestimate the severity of contamination from everyday objects, positively predicted ZIKV anxiety.^34^ Although it is important to stress the importance of ZIKV prevention, these communications must avoid creating undue anxiety and must aim to provide measured recommendations based on ZIKV transmission probabilities in travel destinations and pregnancy intentions. Targeted communication between public health officials or providers about the risks of ZIKV and its sequelae could generate interest in a prophylactic ZIKV vaccine to prevent harmful long-term effects on infected individuals and their infants.

## Supplementary Material

Supplemental Appendix
