# Supplementary material for: United States Travelers’ Concern about Zika Infection and Willingness to Receive a Hypothetical Zika Vaccine
Source: Am J Trop Med Hyg. 2018 Apr 23;98(6):1848–56. doi: 10.4269/ajtmh.17-0907 (PMC6086173; doi:10.4269/ajtmh.17-0907)
Supplement: Supplementary file 1 [file tpmd170907.SD1.pdf]

SUPPLEMENTAL APPENDIX

ZIKA VIRUS KNOWLEDGE QUESTIONS

Zika virus (ZIKV) transmission.

To the best of your knowledge, by which of the following ways can someone get ZIKV? (Correct statements in bold)

A. Receiving a transfusion with blood that contains ZIKV.
B. Breathing the same air as a person who is sick from ZIKV.
C. Being bitten by a mosquito that carries ZIKV.
D. Having sex with someone who is infected with ZIKV.
E. During pregnancy, ZIKV can be passed from a pregnant woman to her fetus (developing baby).
F. Drinking unclean water.
G. Do not know.

ZIKV symptoms.

Which of the following are common symptoms of ZIKV? (Correct symptoms in bold)

A. Mild fever
B. Bloody cough
C. Rash
D. Joint and muscle pain
E. Confusion
F. Pink eye
G. Headache
H. Diarrhea
I. Do not know

ZIKV in pregnancy.

To the best of your knowledge, which of the following statements are true? (Correct statements in bold)

A. Women infected with ZIKV during pregnancy are more likely to have a baby with birth defects than women who have not been infected with ZIKV.
B. Pregnant women who have visited a ZIKV-infected area should be tested for ZIKV whether or not they have symptoms.
C. Public health officials say that insecticides containing DEET are safe for pregnant women to use.
D. ZIKV cannot be transmitted by sex if a woman is pregnant.
E. Do not know.
